# Supplementary material for: Induction of Multiple Immune Signaling Pathways in Gryllodes sigillatus Crickets during Overt Viral Infections
Source: Viruses. 2022 Dec 3;14(12):2712. doi: 10.3390/v14122712 (PMC9786821; doi:10.3390/v14122712)
Supplement: Supplementary file 1 [file viruses-14-02712-s001.zip › viruses-1935942-supplementary.pdf]

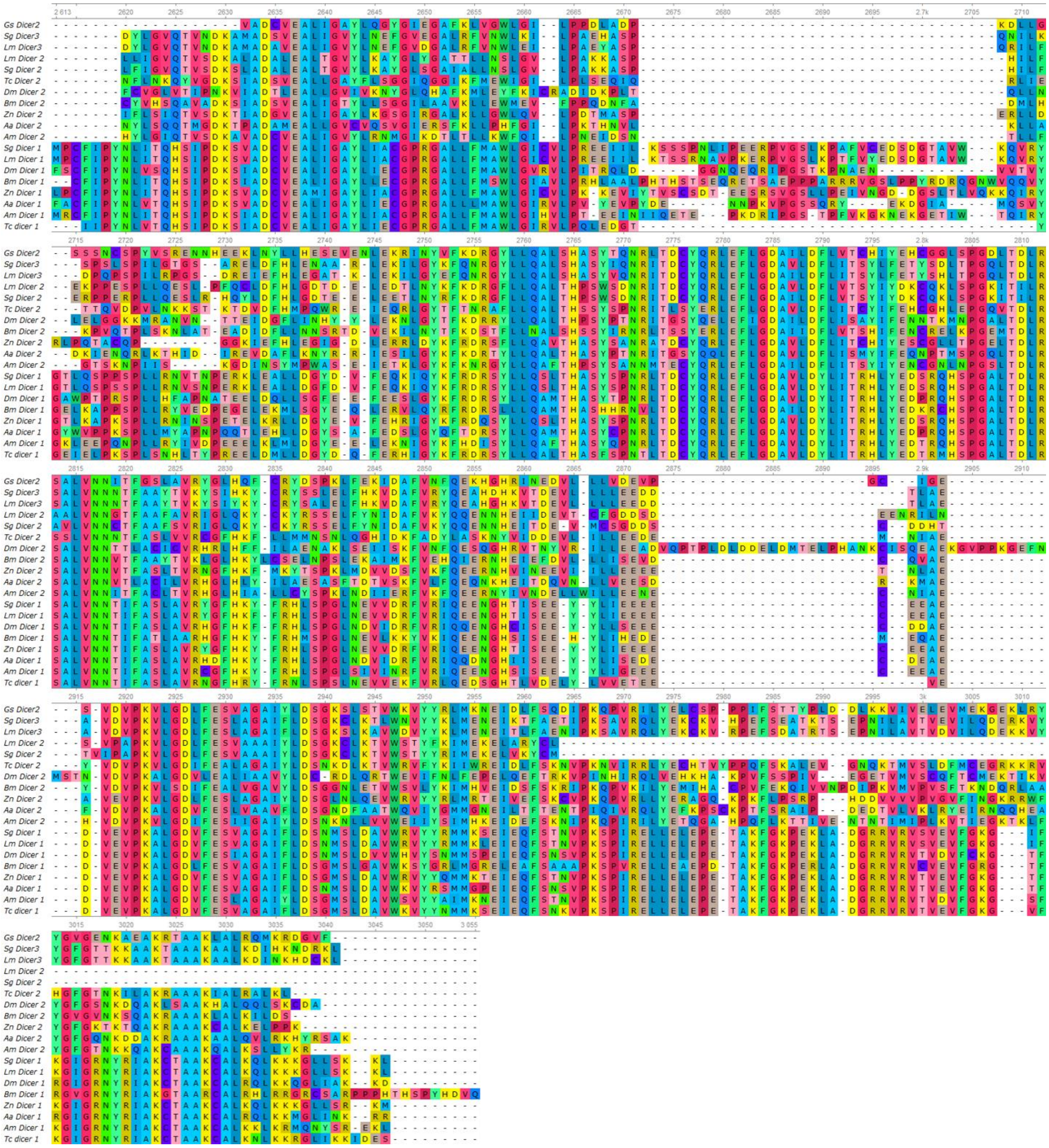

Figure S1: Partial protein alignment of Dicer-2. The partial sequence of *G. sigillatus* Dicer-2 was aligned with Dicer-2 and Dicer 1 sequences from a representative set of insects. Gs = *Gryllodes sigillatus*, Sg: *Schistocerca gregaria*, Lm: *Locusta migratoria*, Tc: *Tribolium castaneum*, Dm: *Drosophila melanogaster*, Bm: *Bombyx mori*, Zn: *Zootermopsis nevadensis*, Aa: *Aedes aegypti*, Am: *Apis mellifera.* All proteins were obtained from the ncbi protein database, and represent the following accession numbers: Sg Dicer3: BAX36479.1, Sg Dicer2: QVD39336.1, Sg Dicer1: BAX36477.1, Lm Dicer3: BAW35366.1, Lm Dicer1: BAW35364.1, Lm Dicer2: BAW35365.1, Dm Dicer1: NP_524453.1, Dm Dicer2: NP_001286540.1, Bm Dicer2: NP_001180543.1, Bm Dicer1: XP_037869731.1, Zn Dicer1: XP_021929007.1, Zn Dicer2: XP_021927098.1, Aa Dicer2: QXE98924.1, Aa Dicer1: XP_001659747.2,

Am Dicer1: NP_001116485.2, Am Dicer2: XP_016773223.2, Tc Dicer1: EFA11550.2, Tc Dicer2: NP_001107840.1.

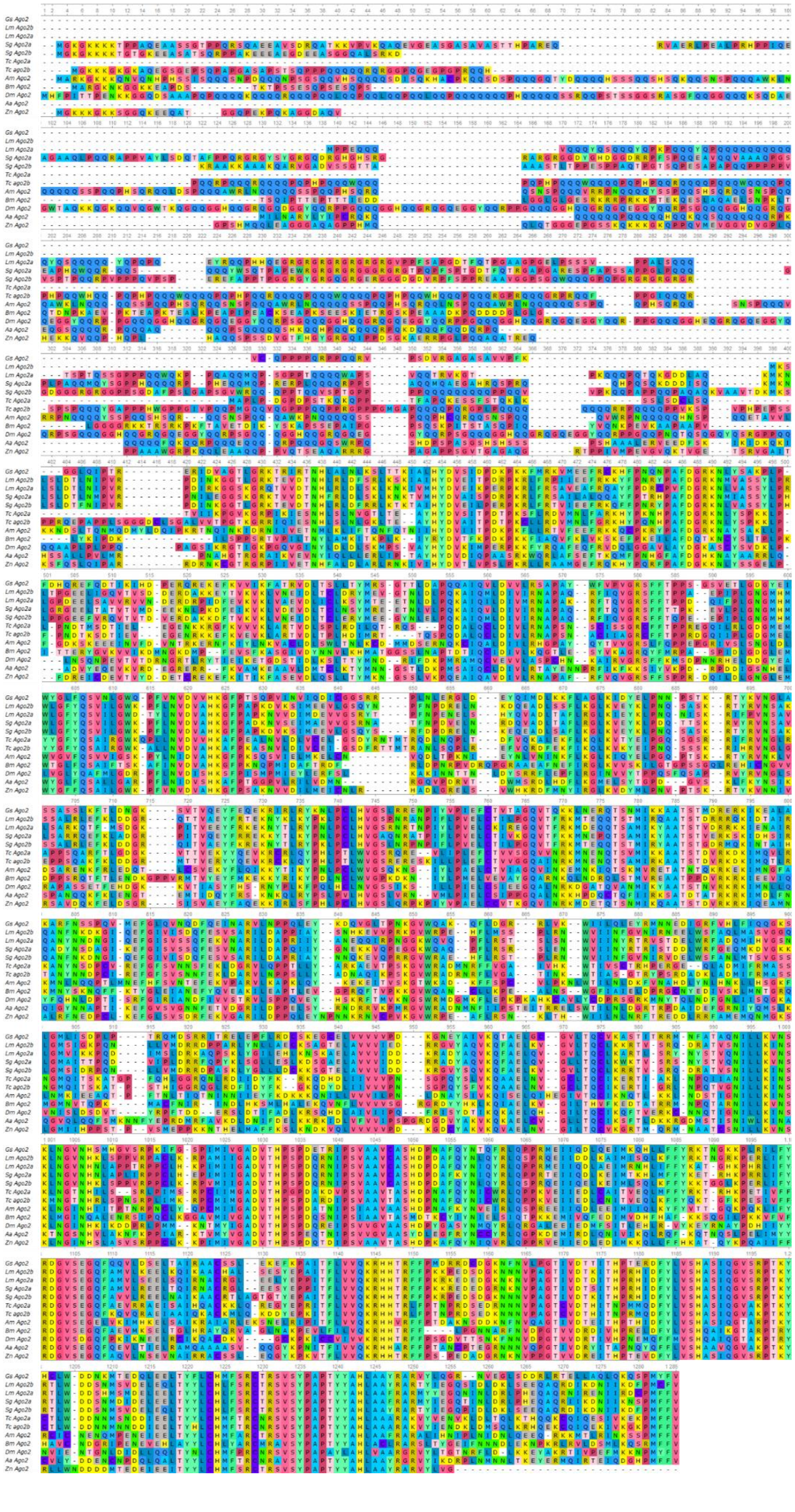
Gs Ago2
Lm Ago2b
Lm Ago2a
Sg Ago2a
Sg Ago2b
Tc Ago2a
Tc ago2b
Am Ago2
Bm Ago2
Dm Ago2
Aa Ago2
Zn Ago2

Figure S2: Protein alignment of Argonaute-2. The sequence of *G. sigillatus* Argonaute-2 was aligned with homologous sequences from a representative set of insects. Gs = *Gryllodes sigillatus*, Sg: *Schistocerca gregaria*, Lm: *Locusta migratoria*, Tc: *Tribolium castaneum*, Dm: *Drosophila melanogaster*, Bm: *Bombyx mori*, Zn: *Zootermopsis nevadensis*, Aa: *Aedes aegypti*, Am: *Apis mellifera.* All proteins were obtained from the ncbi protein database, and represent the following accession numbers: Lm Ago2b: QUP51772.1, Lm Ago2a: QUP51773.1, Sg Ago2b: QVD39218.1, Sg Ago2a: QVD39219.1, Tc Ago2a: EFA11590.1, Tc Ago2b: EFA04626.2, Am Ago2: XP_395048.4, Bm Ago2: NP_001036995.2, Dm Ago2: NP_730054.1, Aa Ago2: XP_011493002.2, Zn Ago2: XP_021934925.1

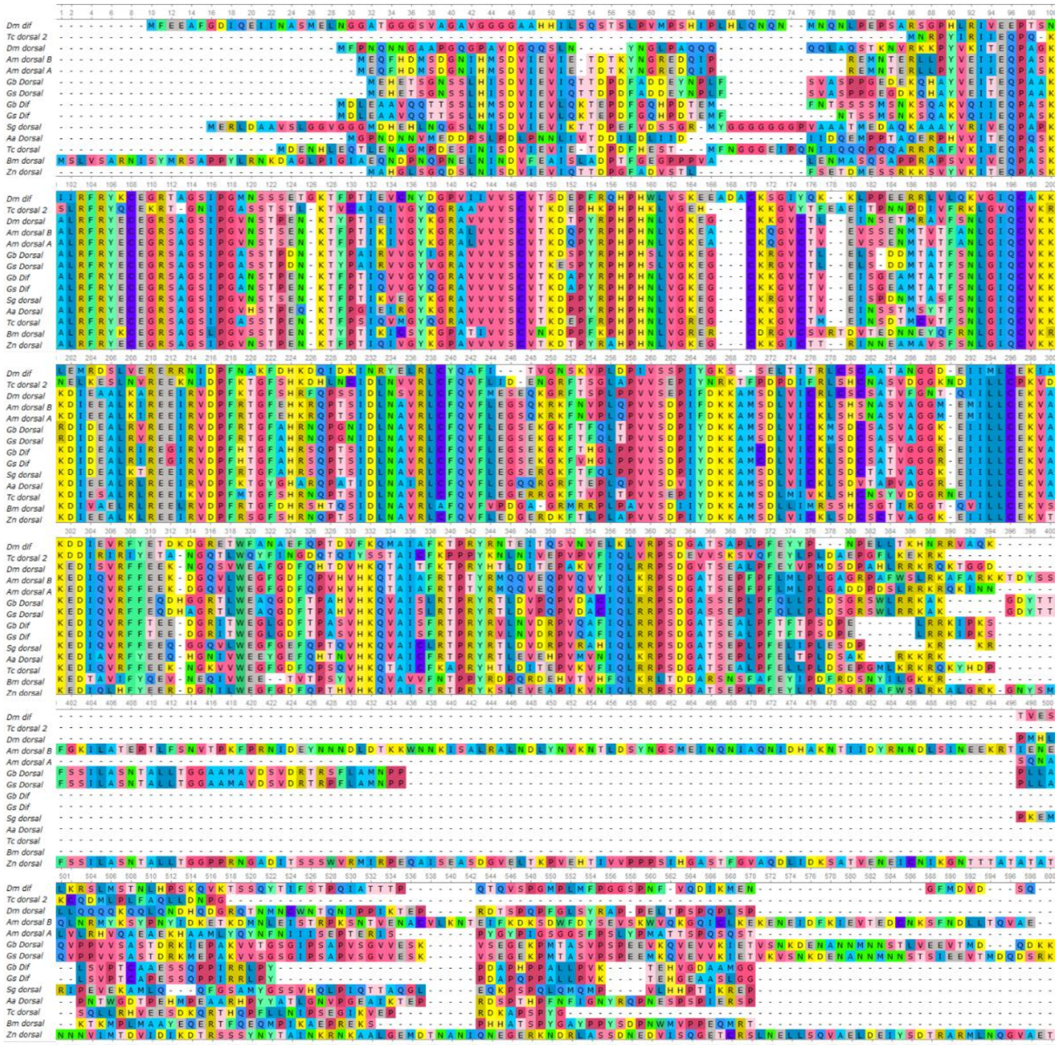
Dm dif
Tc dorsal 2
Dm dorsal
Am dorsal B
Am dorsal A
Gb Dorsal
Gs Dorsal
Gb Dif
Gs Dif
Sg dorsal
Aa Dorsal
Tc dorsal
Bm dorsal
Zn dorsal

Figure S3: Protein alignment of Dorsal. The sequences of *G. sigillatus* Dorsal and Dif were aligned with homologous sequences from a representative set of insects. Gs = *Gryllodes sigillatus*, Gb: *Gryllus bimaculatus*, Sg: *Schistocerca gregaria*, Tc: *Tribolium castaneum*, Dm: *Drosophila melanogaster*, Bm: *Bombyx mori*, Zn: *Zootermopsis nevadensis*, Aa: *Aedes aegypti*, Am: *Apis mellifera.* All proteins were obtained from the ncbi protein database, and represent the following accession numbers: Dm Dorsal: NP_724052.1, Dm Dif: AAA28465.1, Am Dorsal B: NP_001164477.1, Am Dorsal A: NP_001011577.1, Gb Dorsal: BBG28463.1, Gb Dif: BBG28464.1, Sg Dorsal: XP_049833658.1, Aa Dorsal: AAW67214.1, Tc Dorsal 2: EFA02885.1, Tc Dorsal: EFA02850.1, Bm Dorsal: XP_037870194.1, Zn Dorsal: KDR23857.1

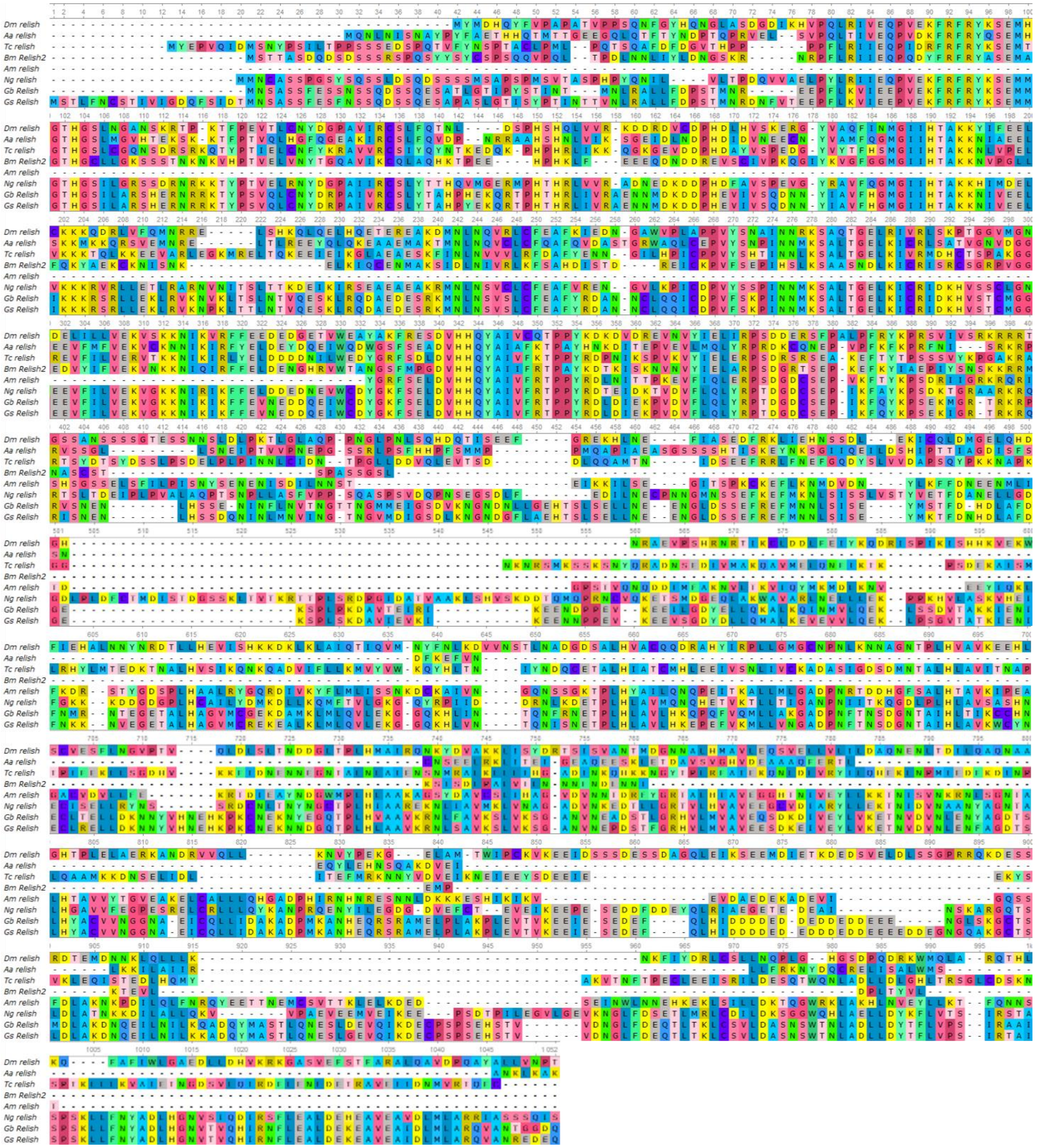

Figure S4: Protein alignment of Relish. The sequences of *G. sigillatus* Relish were aligned with homologous sequences from a representative set of insects. Gs = *Gryllodes sigillatus*, Gb: *Gryllus bimaculatus*, Tc: *Tribolium castaneum*, Dm: *Drosophila melanogaster*, Bm: *Bombyx mori*, Aa: *Aedes aegypti*, Am: *Apis mellifera,* Ns: *Nasutitermes graveolus.* All proteins were obtained from the ncbi protein database, and represent the following accession numbers: Ns Relish: AAZ08468.1, Bm Relish2: BAF74126.1, Tc Relish: EEZ97717.1, Aa Relish: AAM97894.1, Dm Relish: NP_996187.1, Am Relish: ACT66913.1, Gb Relish: BBG28465.1

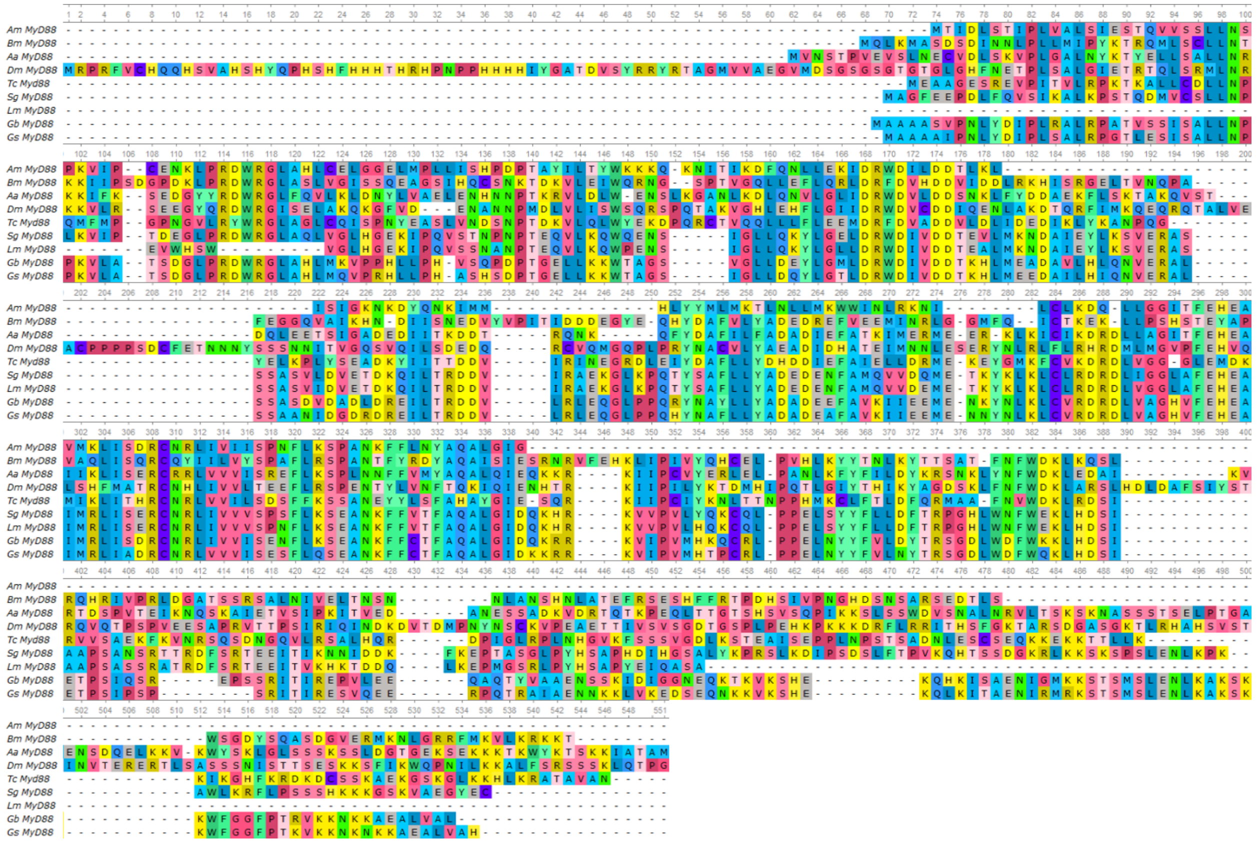

Figure S5: Protein alignment of MyD88. The sequence of *G. sigillatus* MyD88 was aligned with homologous sequences from a representative set of insects. Gs = *Gryllodes sigillatus*, Gb: *Gryllus bimaculatus*, Sg: *Schistocerca gregaria*, Lm: *Locusta migratoria*, Tc: *Tribolium castaneum*, Dm: *Drosophila melanogaster*, Bm: *Bombyx mori*, Aa: *Aedes aegypti*, Am: *Apis mellifera.* All proteins were obtained from the ncbi protein database, and represent the following accession numbers: Am MyD88: AGM19319.1, Tc MyD88: EFA01304.1, Bm MyD88: XP_004921573.1, Sg MyD88: XP_049839790.1, Lm MyD88: AWJ58420.1, Gb MyD88: BBG28459.1, Aa MyD88: XP_001658635.1, Dm MyD88: NP_610479.1

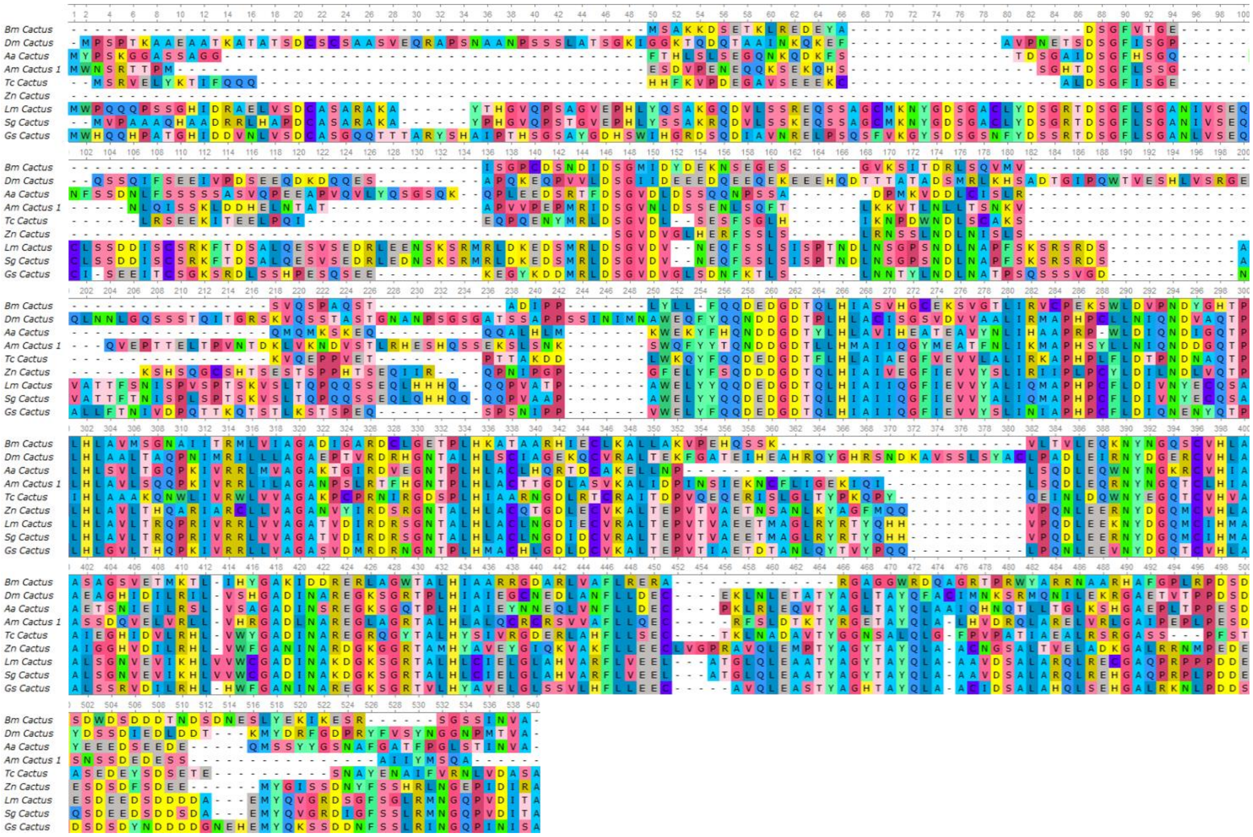

Figure S6: Protein alignment of Cactus. The sequence of *G. sigillatus* Cactus was aligned with homologous sequences from a representative set of insects. Gs = *Gryllodes sigillatus*, Sg: *Schistocerca gregaria*, Lm: *Locusta migratoria*, Tc: *Tribolium castaneum*, Zn: *Zootermopsis nevadensis*, Dm: *Drosophila melanogaster*, Bm: *Bombyx mori*, Aa: *Aedes aegypti*, Am: *Apis mellifera.* All proteins were obtained from the ncbi protein database, and represent the following accession numbers: Am Cactus: NP_001157184.1, Dm Cactus: AAA85908.1, Bm Cactus: BAI67121.1, Lm Cactus: AZP56667.1, Sg Cactus: XP_049833628.1, Aa Cactus: XP_001650267.3, Tc Cactus: KYB24622.1, Zn Cactus: XP_021919362.1

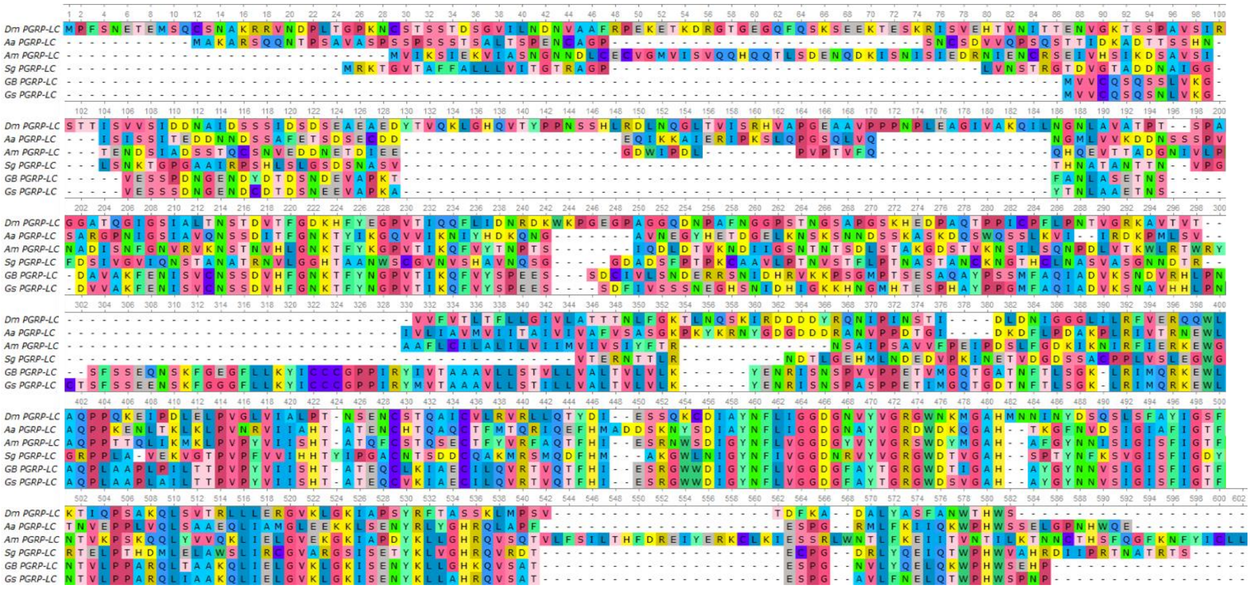

Figure S7: Protein alignment of PGRP-LC. The sequence of *G. sigillatus* PGRP-LC was aligned with homologous sequences from a representative set of insects. Gs = *Gryllodes sigillatus*, Sg: *Schistocerca gregaria*, Gb: *Gryllus bimaculatus*, Dm: *Drosophila melanogaster*, Aa: *Aedes aegypti*, Am: *Apis mellifera.* All proteins were obtained from the ncbi protein database, and represent the following accession numbers: Dm PGRP-LC: NP_648298.1, Am PGRP-LC: KAG9434435.1, Gb PGRP-LC: BBG28440.1, Sg PGRP-LC: XP_049839352.1, Aa PGRP-LC: XP_021698612.1

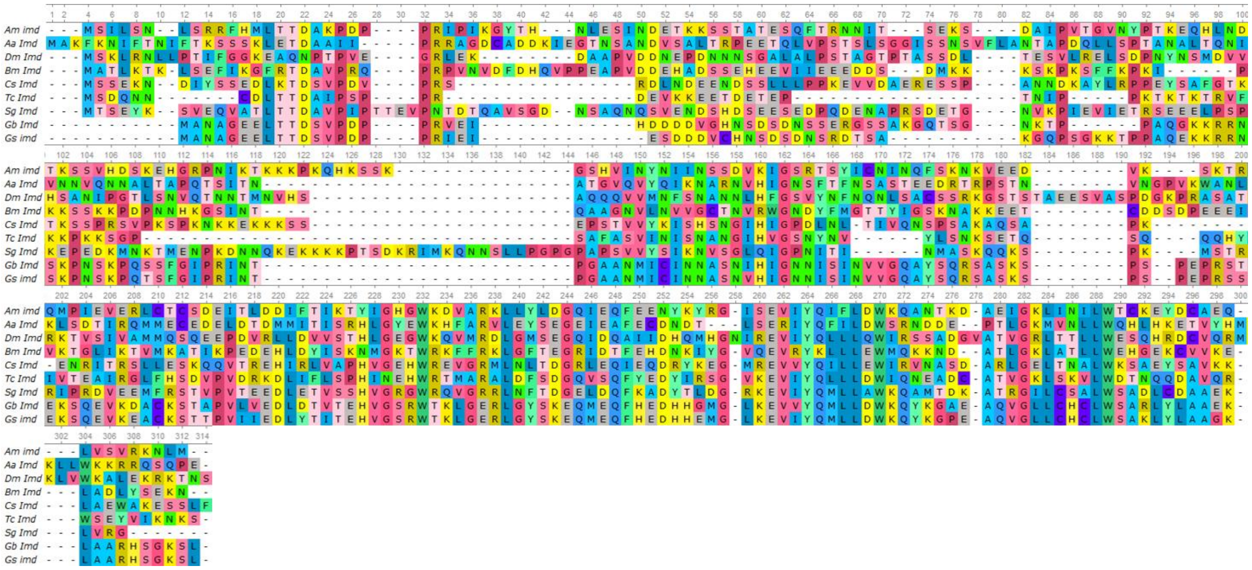

Figure S8: Protein alignment of Imd

The sequence of *G. sigillatus* Imd was aligned with homologous sequences from a representative set of insects. Gs = *Gryllodes sigillatus*, Sg: *Schistocerca gregaria*, Gb: *Gryllus bimaculatus*, Cs: *Cryptotermes secundus*, Tc: *Tribolium castaneum*, Dm: *Drosophila melanogaster*, Bm: *Bombyx mori*, Aa: *Aedes aegypti*, Am: *Apis mellifera.* All proteins were obtained from the ncbi protein database, and represent the following accession numbers: Gb Imd: BBG28437.1, Sg Imd: AFK75938.1, Am Imd: NP_001157189.2, Bm Imd: XP_037866830.1, Cs Imd: XP_023727856.1, Dm Imd: NP_573394.1, Aa Imd: EAT37980.1, Tc Imd: EFA11587.1

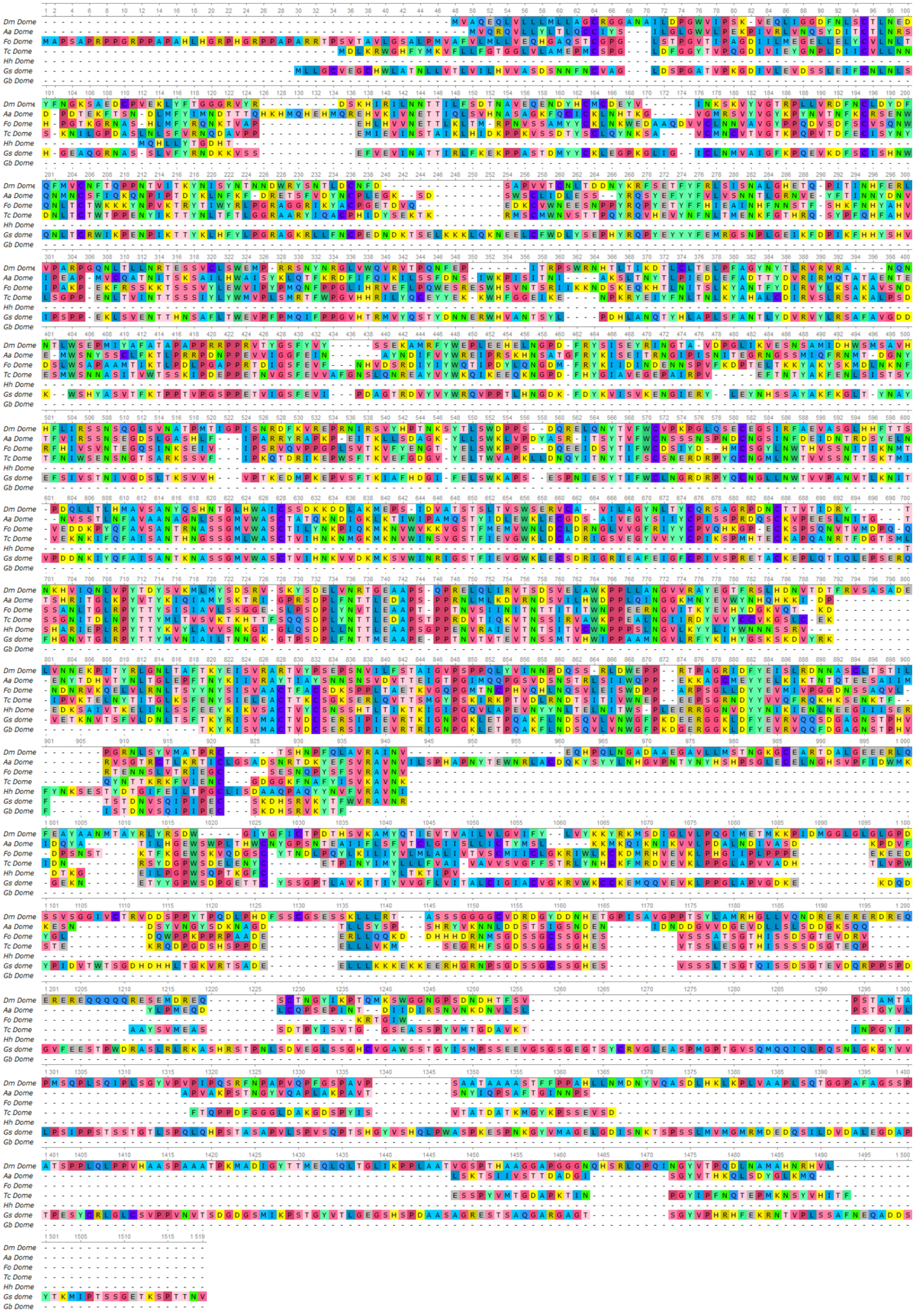
Dm Dome
Aa Dome
Fo Dome
Tc Dome
Hh Dome
Gs dome
Gb Dome

Figure S9: Protein alignment of Domeless

The sequence of *G. sigillatus* Domeless was aligned with homologous sequences from a representative set of insects. Gs = *Gryllodes sigillatus*, Gb: *Gryllus bimaculatus*, Tc: *Tribolium castaneum*, Dm: *Drosophila melanogaster*, Aa: *Aedes aegypti,* Fo: *Frankliniella occidentalis,* Hh: *Halyomorpha halys.* All proteins were obtained from the ncbi protein database, and represent the following accession numbers: Dm Dome: NP_523412.1, Aa Dome: EAT35350.1, Gb Dome: BAN10236.1, Tc Dome: EFA12822.1, Fo Dome: KAE8744265.1, Hh Dome: KAE8573886.1

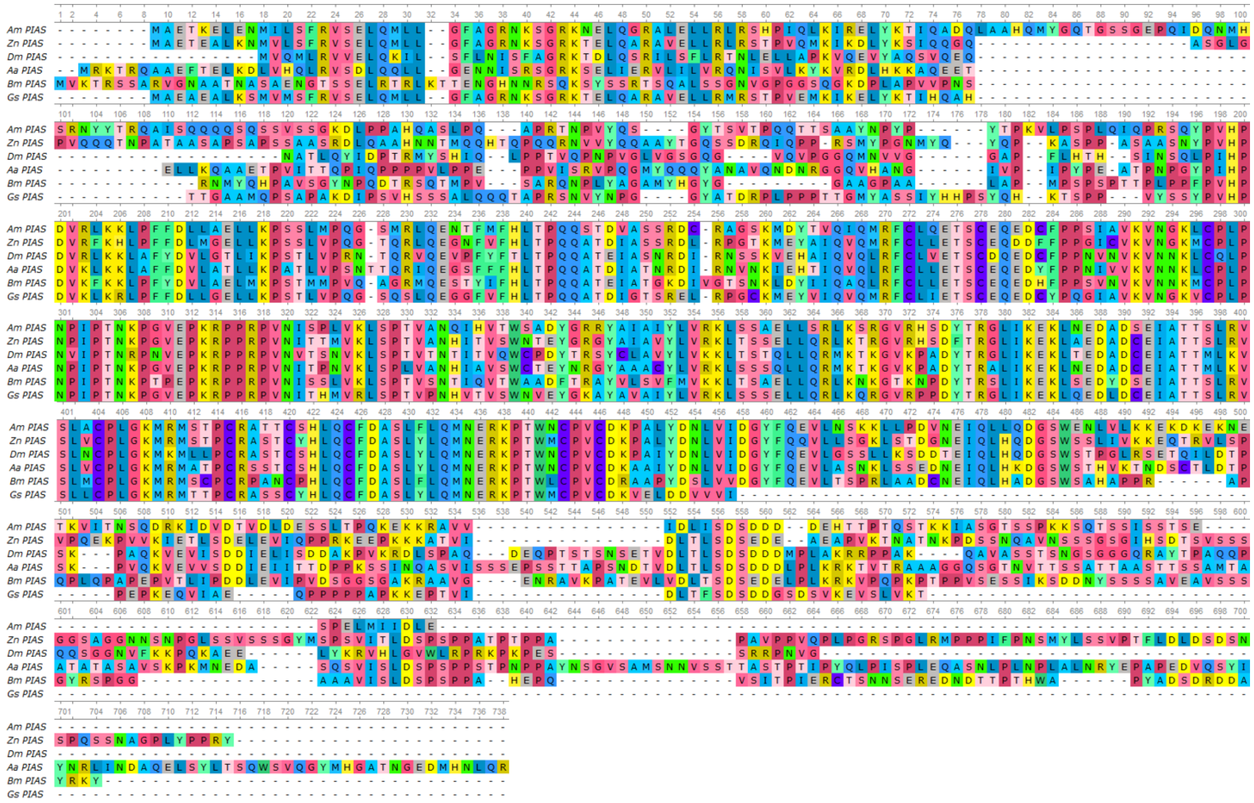

Figure S10: Protein alignment of PIAS

The sequence of *G. sigillatus* PIAS was aligned with homologous sequences from a representative set of insects. Gs = *Gryllodes sigillatus*, Zn: *Zootermopsis nevadensis*, Dm: *Drosophila melanogaster*, Bm: *Bombyx mori*, Aa: *Aedes aegypti*, Am: *Apis mellifera.* All proteins were obtained from the ncbi protein database, and represent the following accession numbers: Am PIAS: XP_623571.3, Zn PIAS: XP_021928824.1, Dm PIAS: AAF58984.1, Aa PIAS: XP_021700150.1, Bm PIAS: XP_004922440.1, Gb PIAS: BAN10244.1

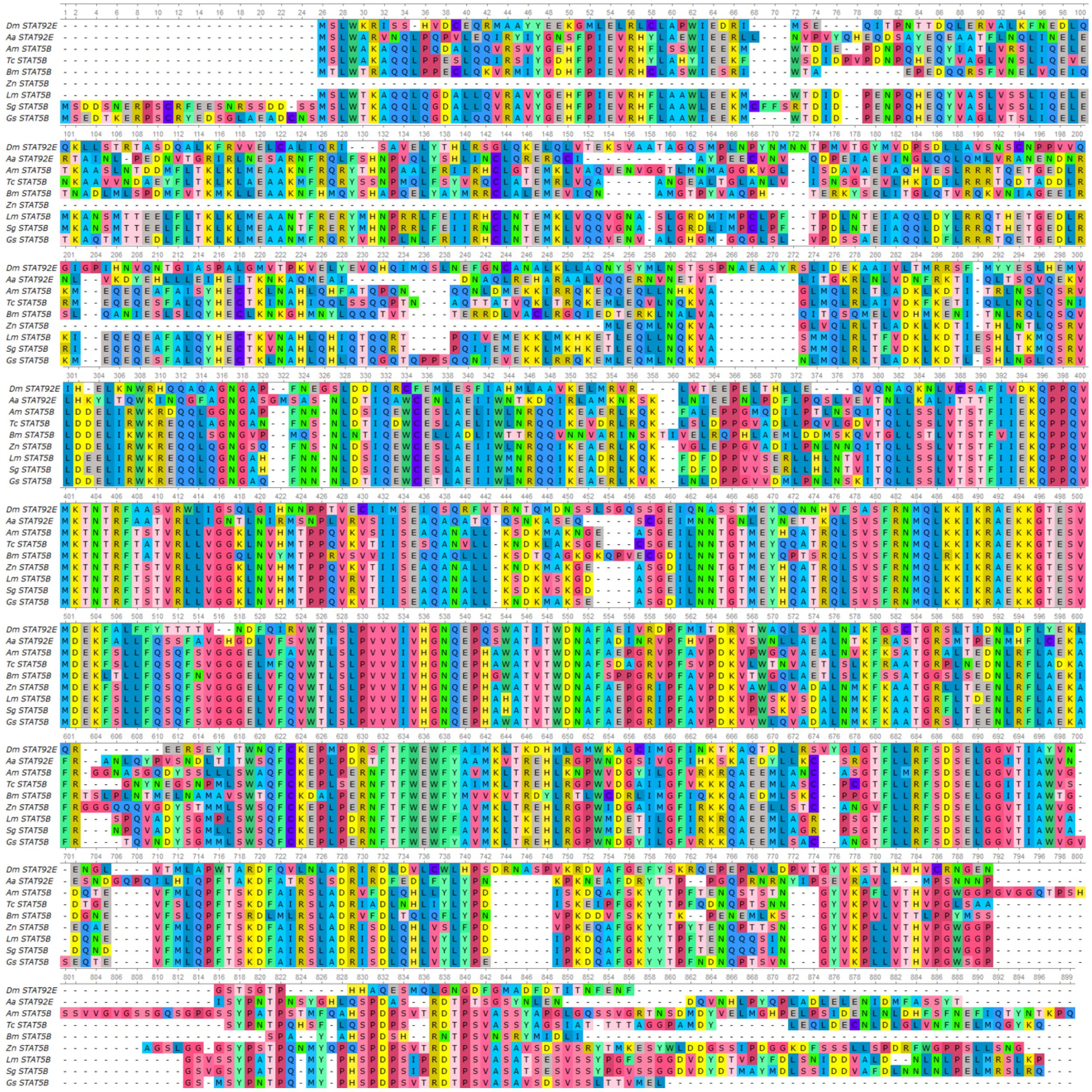

Figure S11: Protein alignment of STAT5B

The sequence of *G. sigillatus* STAT5B was aligned with homologous sequences from a representative set of insects. Gs = *Gryllodes sigillatus*, Sg: *Schistocerca gregaria*, Lm: *Locusta migratoria*, Tc: *Tribolium castaneum*, Zn: *Zootermopsis nevadensis*, Dm: *Drosophila melanogaster*, Bm: *Bombyx mori*, Aa: *Aedes aegypti*, Am: *Apis mellifera.* All proteins were obtained from the ncbi protein database, and represent the following accession numbers: Am STAT5B: XP_397181.1, Lm STAT5B: QTE34030.1, Sg STAT5B: XP_049827783.1, Tc STAT5B: XP_969477.2, Bm STAT5B: NP_001157388.1, Dm STAT92E: AAC46984.1, Aa STAT92E: EAT38424.1, Zn STAT5B: XP_021921231.1

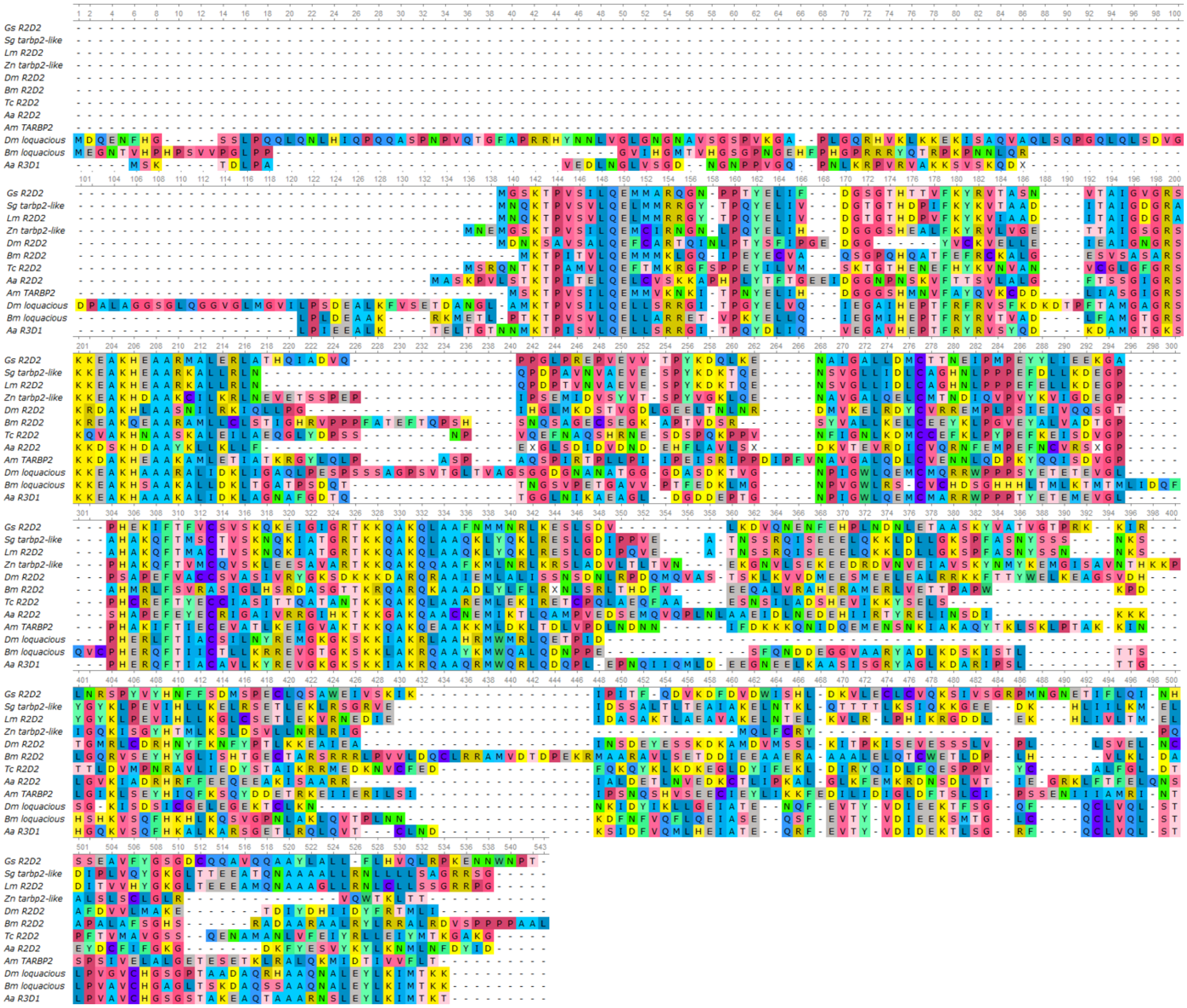

Figure S12: Protein alignment of R2D2

The sequence of *G. sigillatus* R2D2 was aligned with homologous sequences from a representative set of insects. Gs = *Gryllodes sigillatus*, Sg: *Schistocerca gregaria*, Lm: *Locusta migratoria*, Tc: *Tribolium castaneum*, Zn: *Zootermopsis nevadensis*, Dm: *Drosophila melanogaster*, Bm: *Bombyx mori*, Aa: *Aedes aegypti*, Am: *Apis mellifera.* All proteins were obtained from the ncbi protein database, and represent the following accession numbers: Am TARPB2: XP_006560091.1, Sg tarbp2-like: XP_049859483.1, Lm R2D2: UEP55409.1, Zn tarbp2-like: XP_021919678.1, Dm R2D2: ABB77175.1, Bm R2D2: BAJ11654.1, Tc R2D2: NP_001128425.1, Aa R2D2: AJF11544.1, Dm Loquacious: NP_723813.1, Bm Loquacious: NP_001182008.1, Aa R3D1: AJF11546.1

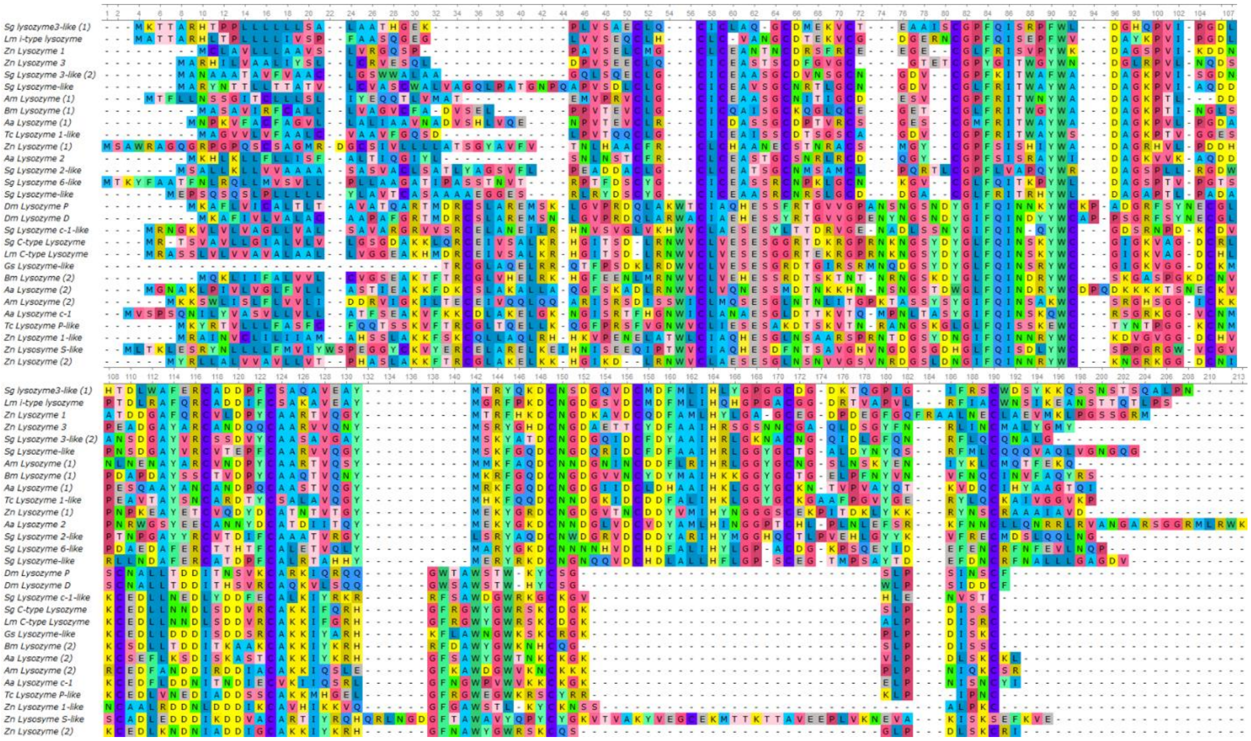

Figure S13: Protein alignment of lysozyme

The sequence of *G. sigillatus* lysozyme was aligned with homologous sequences from a representative set of insects. Gs = *Gryllodes sigillatus*, Sg: *Schistocerca gregaria*, Lm: *Locusta migratoria*, Tc: *Tribolium castaneum*, Zn: *Zootermopsis nevadensis*, Dm: *Drosophila melanogaster*, Bm: *Bombyx mori*, Aa: *Aedes aegypti*, Am: *Apis mellifera.* All proteins were obtained from the ncbi protein database, and represent the following accession numbers: Zn Lysozyme (1): KDR16709.1, Zn Lysozyme (2): KDR20128.1, Zn Lysozyme 1-like: XP_021915960.1, Zn Lysozyme S-like: XP_021915816.1, Zn Lysozyme 1: KDR20129.1, Zn Lysozyme 3: KDR15949.1, Sg C-type Lysozyme: AEX60965.1, Lm c-type Lysozyme: AHE76131.1, Sg Lysozyme c1-like: XP_049831130.1, Sg Lysozyme 6-like: XP_049856590.1, Sg Lysozyme 2-like: XP_049855937.1, Sg Lysozyme 3-like (1): XP_049850555.1, Sg Lysozyme 3-like (1): >XP_049850546.1, Sg Lysozyme-like_0: XP_049856729.1, Sg Lysozyme-like: XP_049850554.1, Lm i-type lysozyme: APD13829.1, Am Lysozyme (1): XP_026300526.1, Am Lysozyme (2) : XP_393161.3, Bm Lysozyme (1): AAB40947.1, Bm Lysozyme (2): XP_004926302.1, Aa Lysozyme (1): AAU09087.1, Aa Lysozyme (2): XP_021706771.1, Aa Lysozyme c-1: XP_021696194.1, Aa Lysozyme 2: XP_001653399.1, Dm Lysozyme P: NP_476828.1, Dm Lysozyme D: NP_476823.1, Tc Lysozyme 1-like: KYB26736.1, Tc Lysozyme 1-like: KYB26179.1

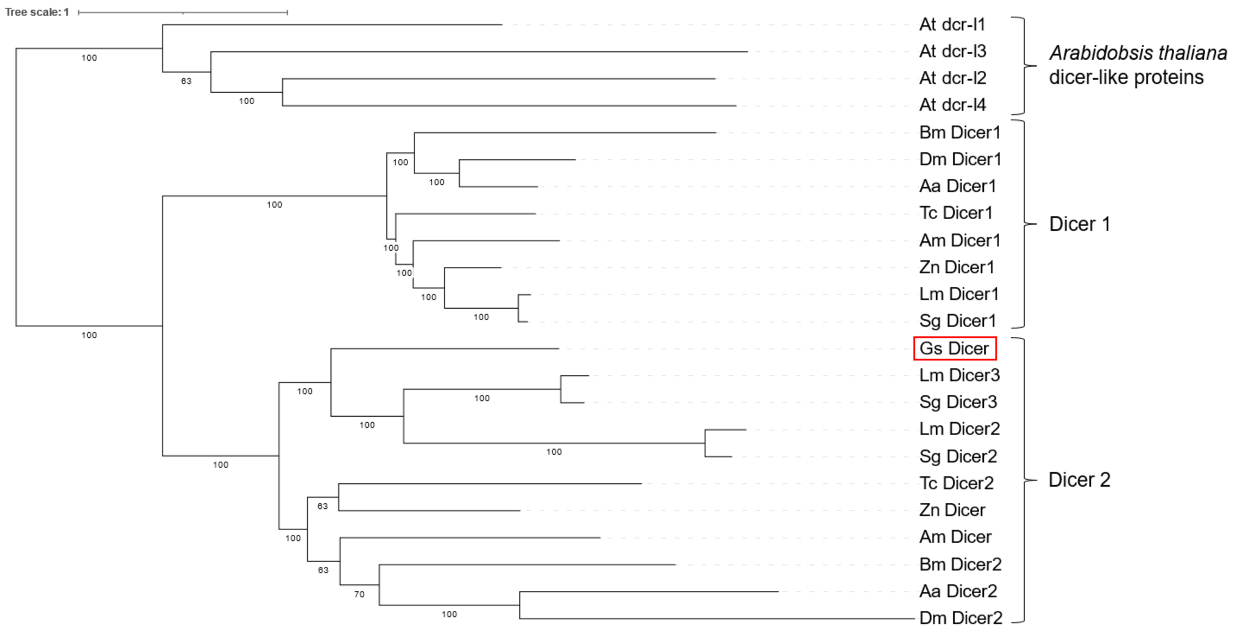

Figure S14: Maximum likelihood tree of Dicer proteins. A Maximum Likelihood tree of Dicer proteins from a representative set of insects is shown, showing the position of *G. sigillatus* Dicer-2 within the clade of Dicer 2 proteins. Bootstrap support values above 90, obtained from 1000 bootstraps, were added to each branch. Gs = *Gryllodes sigillatus*, Sg: *Schistocerca gregaria*, Lm: *Locusta migratoria*, Tc: *Tribolium castaneum*, Dm: *Drosophila melanogaster*, Bm: *Bombyx mori*, Zn: *Zootermopsis nevadensis*, Aa: *Aedes aegypti*, Am: *Apis mellifera,* At: *Arabidopsis thaliana.* All proteins were obtained from the ncbi protein database, and represent the following accession numbers: Sg Dicer3: BAX36479.1, Sg Dicer2: QVD39336.1, Sg Dicer1: BAX36477.1, Lm Dicer3: BAW35366.1, Lm Dicer1: BAW35364.1, Lm Dicer2: BAW35365.1, Dm Dicer1: NP_524453.1, Dm Dicer2: NP_001286540.1, Bm Dicer2: NP_001180543.1, Bm Dicer1: XP_037869731.1, Zn Dicer1: XP_021929007.1, Zn Dicer2: XP_021927098.1, Aa Dicer2: QXE98924.1, Aa Dicer1: XP_001659747.2, Am Dicer1: NP_001116485.2, Am Dicer2: XP_016773223.2, Tc Dicer1: EFA11550.2, Tc Dicer2: NP_001107840.1, At dcr-l1: NP_171612.1, At dcr-l2: AEE73925.1, At dcr-l3: AEE77844.1, At dcr-l4: NP_001190348.1.

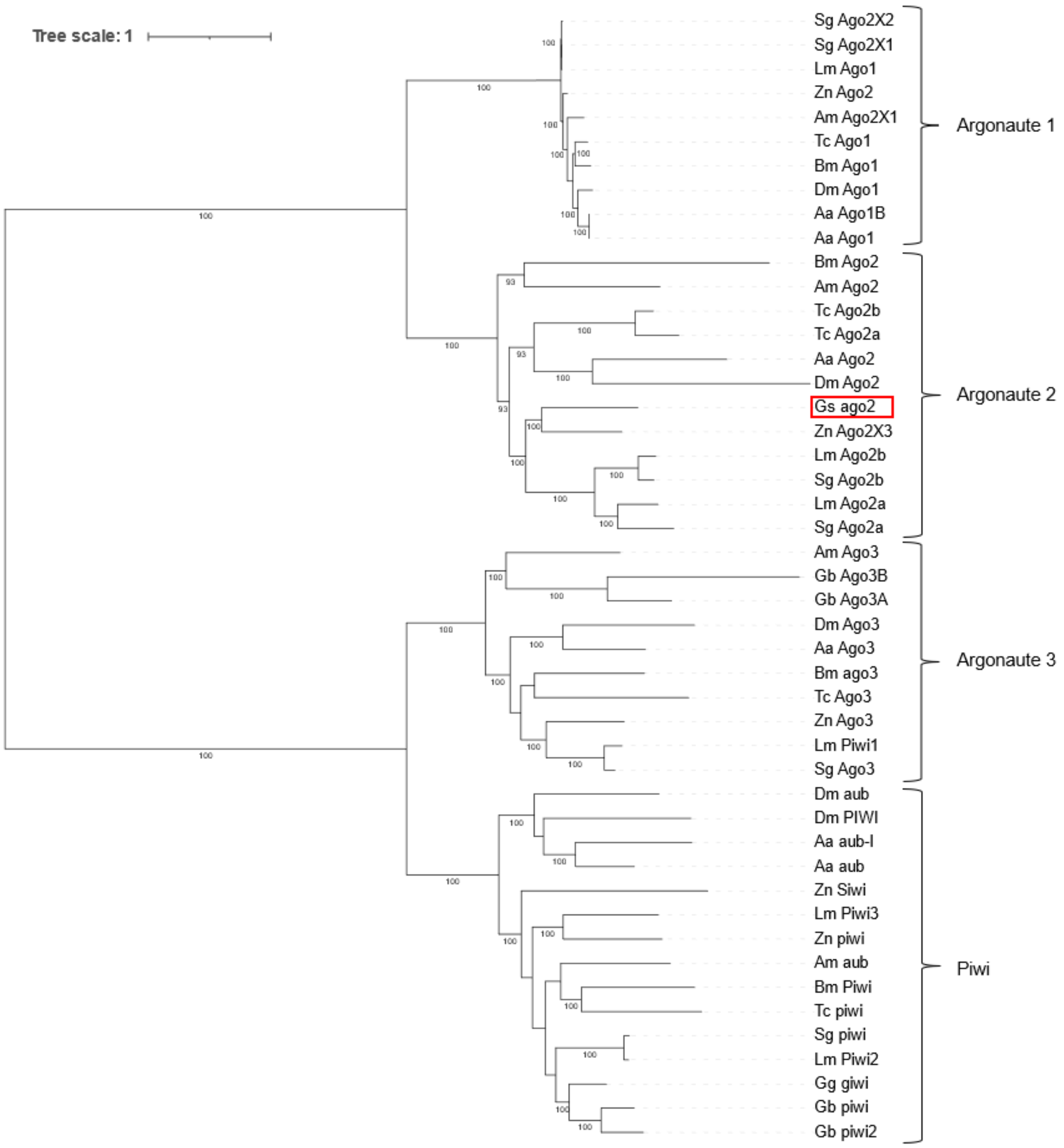

Figure S15: Maximum likelihood tree of Argonaute and piwi proteins. A Maximum Likelihood tree of Argonaute and piwi proteins from a representative set of insects is shown, showing the position of *G. sigillatus* Argonaute-2 within the clade of Argonaute-2 proteins. Bootstrap support values above 90, obtained from 1000 bootstraps, were added to each branch. Gs = *Gryllodes sigillatus*, Sg: *Schistocerca gregaria*, Lm: *Locusta migratoria*, Tc: *Tribolium castaneum*, Dm: *Drosophila melanogaster*, Bm: *Bombyx mori*, Zn: *Zootermopsis nevadensis*, Aa: *Aedes aegypti*, Am: *Apis mellifera,* Gg: *Gampsocleis gratiosa.* All proteins were obtained from the ncbi protein database, and represent the following accession numbers: Lm Ago2b: QUP51772.1, Lm Ago2a: QUP51773.1, Sg Ago2b: QVD39218.1, Sg Ago2a: QVD39219.1, Tc Ago2a: EFA11590.1, Tc Ago2b: EFA04626.2, Am Ago2: XP_395048.4, Bm Ago2: NP_001036995.2, Dm Ago2: NP_730054.1, Aa Ago2: XP_011493002.2, Zn Ago2: XP_021934925.1, Lm Piwi3: BAW35371.1, Lm Piwi2: BAW35370.1, Lm Piwi1: BAW35369.1, Lm Ago1: BAW35367.1, Sg Ago2X2: XP_049830024.1, Sg Ago2X1: XP_049830023.1, Sg Siwi: XP_049864520.1, Sg Ago3: XP_049857113.1, Gb piwi: AFV31611.1, Gb piwi2: AGJ83080.1, Gg giwi: AFW04603.1, Gb Ago3A: AGJ83081.1, Gb Ago3B: AGJ83082.1, Tc Ago1: EFA09197.2, Tc Ago3: EFA02921.1, Tc piwi: EFA07425.1, Am Ago2X1: XP_026300412.1, Am aub: XP_026300661.1, Am Ago3: XP_026300549.1, Bm Ago3: BAF73717.2, Bm

Ago1: NP_001095931.1, Bm Piwi: BAF98574.1, Dm Ago1: NP_725341.1, Dm Ago3: NP_001036628.2, Dm PIWI: AAD08704.1, Dm aub: NP_476734.1, Aa Ago1B: EAT32599.1, Aa Ago1: QPA18337.1, Aa Ago3: XP_001652945.1, Aa aub: XP_001653082.1, Aa aub-l: XP_021712346.1, Zn piwi: KDR24114.1, Zn Siwi: XP_021936690.1, Zn Ago3: XP_021933458.1.

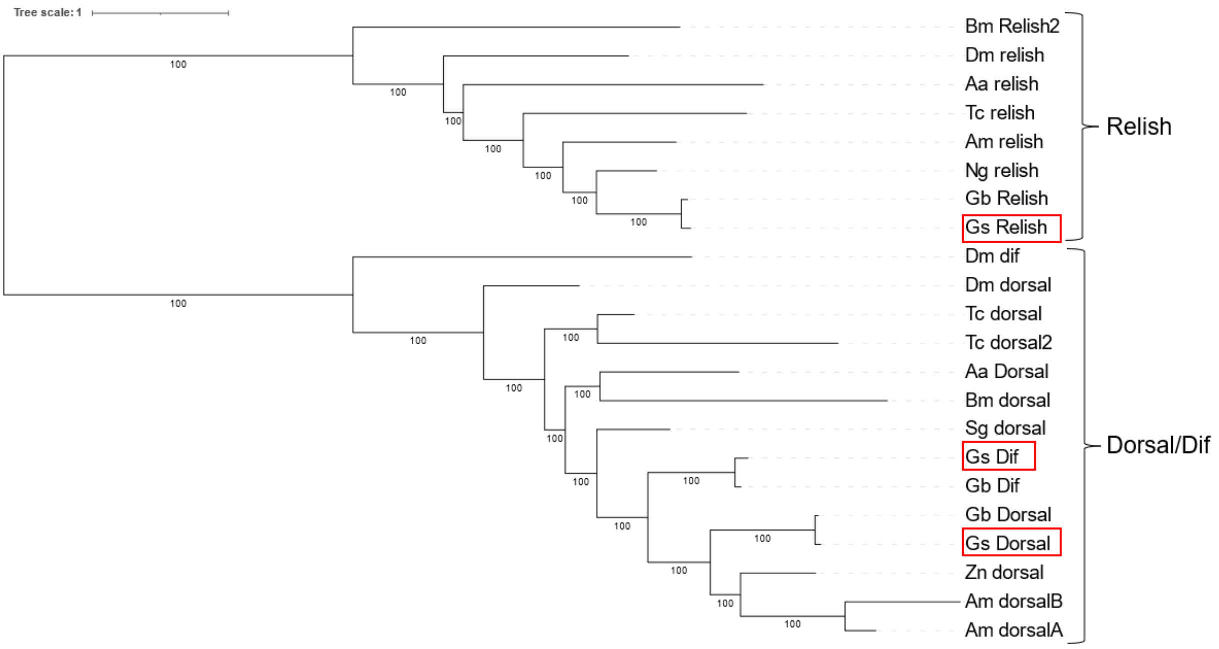

Figure S16: Maximum likelihood tree of Dorsal and Relish proteins. A Maximum Likelihood tree of Dorsal and Relish proteins from a representative set of insects is shown. Bootstrap support values above 90, obtained from 1000 bootstraps, were added to each branch. Gs = *Gryllodes sigillatus*, Gb: *Gryllus bimaculatus*, Sg: *Schistocerca gregaria*, Tc: *Tribolium castaneum*, Dm: *Drosophila melanogaster*, Bm: *Bombyx mori*, Zn: *Zootermopsis nevadensis,* Ns: *Nasutitermes graveolus*, Aa: *Aedes aegypti*, Am: *Apis mellifera.* All proteins were obtained from the ncbi protein database, and represent the following accession numbers: Dm Dorsal: NP_724052.1, Dm Dif: AAA28465.1, Am Dorsal B: NP_001164477.1, Am Dorsal A: NP_001011577.1, Gb Dorsal: BBG28463.1, Gb Dif: BBG28464.1, Sg Dorsal: XP_049833658.1, Aa Dorsal: AAW67214.1, Tc Dorsal 2: EFA02885.1, Tc Dorsal: EFA02850.1, Bm Dorsal: XP_037870194.1, Zn Dorsal: KDR23857.1, Ns Relish: AAZ08468.1, Bm Relish2: BAF74126.1, Tc Relish: EEZ97717.1, Aa Relish: AAM97894.1, Dm Relish: NP_996187.1, Am Relish: ACT66913.1, Gb Relish: BBG28465.1
